# The Function and Expression of ATP-Binding Cassette Transporters Proteins in the Alzheimer's Disease

**DOI:** 10.1055/s-0041-1735541

**Published:** 2021-09-17

**Authors:** Asli Aykac, Ahmet Özer Sehirli

**Affiliations:** 1 Department of Biophysics, Near East University, Nicosia, Cyprus; 2 Department of Pharmacology, Faculty of Dentistry, Near East University, Nicosia, Cyprus

**Keywords:** ABC transporters, Alzheimer's diseases, amyloid-β, P-glycoprotein, multidrug resistance protein 1

## Abstract

Despite many years of research, radical treatment of Alzheimer's disease (AD) has still not been found. Amyloid-β (Aβ) peptide is known to play an important role in the pathogenesis of this disease. AD is characterized by three main changes occurring in the central nervous system: (1) Aβ plaque accumulation that prevents synaptic communication, (2) the accumulation of hyperphosphorylated tau proteins that inhibit the transport of molecules inside neurons, and (3) neuronal cell loss of the limbic system. Mechanisms leading to Aβ accumulation in AD are excessive Aβ production as a result of mutations in amyloid precursor protein or genes, and impairment of clearance of Aβ due to changes in Aβ aggregation properties and/or Aβ removal processes. Human ATP-binding cassette (ABC) transporters are expressed in astrocyte, microglia, neuron, brain capillary endothelial cell, choroid plexus, choroid plexus epithelial cell, and ventricular ependymal cell. ABC transporters have essential detoxification and neuroprotective roles in the brain. The expression and functional changes in ABC transporters contribute to the accumulation of Aβ peptide. In conclusion, the review was aimed to summarize and highlight accumulated evidence in the literature focusing on the changing functions of human ABC transporter members, in AD pathogenesis and progression.

## Introduction

Central nervous system (CNS) barriers, consisting of the blood–brain barrier (BBB) and blood–cerebrospinal fluid barrier, are responsible for the protection of the microenvironment, which is vital in the regulation of neuronal functions. 1 There is increasing evidence that abnormal expression of efflux transporters (ATP-binding cassette [ABC]) in the BBB, a semipermeable barrier composed of endothelial cells, causes CNS-related diseases such as Parkinson's disease and Alzheimer's disease (AD). In addition, the dysfunctions that occur in these transporters cause the disruption of the integrity of the BBB, the deterioration of the CNS balance, and consequently, the exacerbation of CNS diseases, including AD. 1

The treatment of millions of patients suffering from AD associated with microvascular dysfunction and/or degeneration is still a mystery despite years of research efforts. Therefore, there is a great need to identify new therapies that target the underlying causes of AD, prevent or eliminate existing symptoms. 2 A dense accumulation of amyloid-β (Aβ), that is, a peptide resulting from amyloid precursor protein (APP) processing, and deposition of hyperphosphorylated tau protein appear in the CNS as AD pathology. 3 4 AD is also closely associated with microvascular pathologies, such as degeneration and/or dysfunction in the microvascular structure, which are key places in the exchange of nutrients in the brain between the brain and circulating blood. These pathologies cause Aβ accumulation by disrupting the integrity of the BBB and preventing the clearance of neurotoxic molecules (such as Aβ) in the CNS. 1 Microtubule-associated tau proteins and the Aβ _40_ , Aβ _42_ , the most common form of Aβ produced by cleavage of APP, represent the molecular-level character of AD. Although studies conducted in the brains of AD patients have determined 100 times more Aβ plaque formation compared with controls, the physiopathological effect of AD is not fully elucidated. Hyperphosphorylation of tau proteins, another molecular marker determined in patients with AD, causes microtubular collapse. 5 The accumulation of these molecules in the brain increases as the neurotoxic molecules, which accumulate due to the deterioration of the BBB balance, are not cleaned in the CNS. One of the families of transporter that can mediate Aβ homeostasis and play key roles in AD pathophysiology is ABC transporters.

Enabling the movement of their substrates through intracellular organelles and cell membranes, ABC transporters, which provide the movement of its substrates from intracellular organelles and cell membranes, ensure the homeostasis of the body. 6 Recently, the emphasis on the role of ABC carriers in CNS disorders associated with high levels of Aβ, such as AD, has increased research into the causes of changes or dysfunction in the processes and pathways in which the carrier family is involved. The contribution of ABC carriers to AD physiopathology is not fully known, and studies on this subject will contribute to the resolution of the relationship between AD process and ABC carriers and accordingly to the development of new treatment strategies. 6 7

AD is characterized by three main changes occurring in the CNS: (1) Aβ plaque accumulation that prevents synaptic communication, (2) the accumulation of hyperphosphorylated tau proteins that inhibit the transport of molecules inside neurons, and (3) neuronal cell loss of the limbic system. 5 The basis of AD is explained by two main theories: (1) the “Aβ cascade hypothesis” emphasizing that it develops with the accumulation of oligomeric Aβ species in the CNS and (2) the “neurovascular hypothesis” that tries to determine the accumulation of Aβ species. According to the neurovascular hypothesis, among the causes of Aβ accumulation in the CNS, the absence or reduction of Aβ degradation, reduction of Aβ clearance, or increase of Aβ influx along the BBB is indicated. 8 Mechanisms leading to Aβ accumulation in AD are excessive Aβ production as a result of mutations in APP or genes, and impairment of clearance of Aβ due to changes in Aβ aggregation properties and/or Aβ removal processes. ABC transporters are responsible for AD physiopathology by preventing direct or indirect Aβ accumulation in the BBB by active transport. 5

In our review, we aimed to evaluate the roles and physiopathological features of human ABC carrier members expressed in the CNS and associated with AD pathogenesis.

## ABC Transporters

Representing the largest family of integral proteins, *ABC* genes use ATP energy to transport various molecules across all cell membranes. 9 ABC proteins are classified according to the sequence and organization of ATP-binding domains. Usually, unidirectional ATP pumps move hydrophobic compounds from the cytoplasm to extracellular or intracellular organelles such as the endoplasmic reticulum and mitochondria. 10 ABC transporters transfer metabolic wastes to the cytoplasm and from the cytoplasm to the blood. ABC transporters also prevent xenobiotics from entering the brain. 1

Genes are divided into subfamilies based on the order of domains similarity in their structure in the nucleotide-binding folds and transmembrane domains. ABC transporters are divided into seven subfamilies and it is known that ∼50 ABC transporters (ABCA1–13, ABCB1–11, ABCC1–6, CFTR, ABCC8–12, ABCD1–4, ABCE1, ABCF1–3, ABCG1,2,4,5,8) are expressed in the human genome. 10 11 Human ABC transporter proteins are localized in many tissues such as brain, liver, intestine, etc. 12 13 Their presence in many tissues and their involvement in many physiological and biochemical processes are an indication of the importance of ABC transporters. Abnormalities or dysfunctions that will occur in these transporters emphasize their relationship with a wide range of diseases, from cystic fibrosis to cancer, especially neurodegenerative diseases. 14

ABC transporters similarly function as a second selective permeability barrier in neurons and glial cells. Because of all of these properties of ABC transporters, they have essential detoxification and neuroprotective roles in the brain. 15 16 Changes in ABC carrier expressions or functions lead to β clearance disturbances, causing AD-associated cerebral amyloid angiopathy (CAA). 17 18 19 20

## ABCA Subfamily

The largest *ABC* gene subfamily is ABCA which consists of a lot of amino acids. 21 ABCA full transporter is divided into two groups: ABCA1 like (ABCA1–4,7,12,13) and ABCA5 like (ABCA5,6,8–10). 11 22 ABCA1 and ABCA2 proteins, which play a role in apolipoprotein-dependent cholesterol efflux, sterol homeostasis, lipid transport, and metabolism, share ∼50% sequence homology. 23 24

*ABCA1* : This transporter is highly expressed in brain cells ( Table 1 ). The fact that ABCA1 mediates the flow of cholesterol from astrocytes and microglia in the CNS facilitates lipidation of ApoE. 25 Cholesterol level constitutes an important component of AD pathogenesis because it regulates the properties of the membrane where enzymes necessary for Aβ production are localized. ApoE, which is an important factor for brain cholesterol homeostasis, provides development, repair, and nerve growth to brain cells. The association of altered cholesterol metabolism in the brain with increased AD is indicative of the critical role that ApoE has in AD. Individuals carrying the ApoE-ε4 alleles, one of the ApoE isoforms, have a greater risk of developing AD because these alleles directly interact with Aβ and promote Aβ aggregation and plaque formation. 26 27 In the AD mouse model overexpressing J20-human APP (hAPP), ABCA1 deficiency was shown to cause reduced lipid flux and a dramatic decrease in brain ApoE levels. With studies in mice overexpressing hAPP, it is shown that ABCA1 deficiency increases Aβ _40_ :Aβ _42_ ratio. 28 A decrease in ABCA1 causes greater accumulation of amyloid, increasing ABCA1 protein or function decreases amyloid accumulation. 4 In the study by Kim et al (2013), it was found that neuronal ABCA1 mRNA and protein levels in the hippocampus of the brains of AD patients were not affected and were significantly upregulated compared with the cerebellum. 4 The authors thought that this upregulation was related to the pathological process of AD. Similar to these findings, it was determined that hippocampal ABCA1 expression was upregulated in an age-dependent manner in the experimental results performed in APP/PS1 mice. It has been reported that cholesterol efflux decreases in astrocytes and microglia in mice with ABCA1 deficiency, resulting in cellular accumulation of cholesterol and decreased ApoE lipidation. Moreover, it has been reported that a decrease in lipidated ApoE level decreases amyloid-proteolytic degradation that increases the risk of AD. 25

**Table 1:** ABC transporters expression in human brain cell and their roles in AD

ABC subtype	Expression	Role
ABCA1	BEC, CP, CPEC, VEC, astrocyte, microglia, oligodendrocyte, neuron	ABCA1 loss, Aβ accumulation in endothelial cells Increased ABCA1 function in astrocytes reduces Aβ accumulation Decreases the influx of Aβ across the BBB
ABCA2	BEC, astrocyte, microglia, oligodendrocyte, neuron	ABCA2 increase APP and Aβ levels
ABCA5	BEC, astrocyte, microglia, oligodendrocyte, neuron	Reduce the formation of Aβ _40_ and Aβ _42_
ABCA7	BEC, astrocyte, microglia, oligodendrocyte, neuron	Inhibits Aβ production plays a role in APP processing leads to enhanced Aβ secretion
ABCB1/P-gp	BEC, CP, CPEC, VEC, astrocyte, microglia, pericyte, oligodendrocyte, neuron	ABCB1 is downregulated in brain endothelial cells in AD Transport the Aβ and also plays a role in clearance the Aβ from the brain
ABCC1	BEC, CP, CPEC, VEC, astrocyte, microglia, pericyte, neuron	Decrease Aβ levels Lack of ABCC1 increase Aβ _40_ and Aβ _42_ levels
ABCG1	BEC, CP, CPEC, astrocyte, microglia, oligodendrocyte, neuron,	Regulate cellular homeostasis and not appear to have direct role AD
ABCG2/BCRP	BEC, astrocyte, microglia, neuron, pericyte	Prevents amyloid accumulation and resting the passage of circulating Aβ
ABCG4	BEC, CP, CPEC, VEC, astrocyte, microglia, neuron	Response to clearance Aβ Suppresses ABCG4 increases Aβ secretion

Abbreviations: Aβ, amyloid-β; ABC, ATP-binding cassette; AD, Alzheimer's disease; APP, amyloid precursor protein; BBB, blood–brain barrier; BEC, brain capillary endothelial cell; CP, choroid plexus; CPEC, choroid plexus epithelial cell; VEC, ventricular ependymal cell.

Note: Pericytes, an essential component of neurovascular unit, also known as BBB gatekeepers, carry nutrients and waste molecules between blood and brain interstitial fluid.

*ABCA2* : ABCA2 is mostly expressed in the hippocampus and especially white matter ( Table 1 ). 29 30 31 The functional studies of ABCA2 show that ABCA2 plays a role in neural transmembrane lipid transport and metabolism. 10 Decreasing the expression of ABCA2 leads to an increase in levels of low-density lipoprotein. In a study comparing ABCA2 expression levels in different brain regions of patients with AD, the highest level of ABCA2 was found in the temporal and frontal regions. 32 These study results emphasize the importance of precisely defining the positive correlation between Aβ and ABCA2 in the pathogenesis of AD. In another study consistent with these findings, it was reported that ABCA2 expression increased in the temporal region in AD patients, and it was determined that the excessive increase in this protein expression was affected by Aβ clearances. Further, unlike ABCA1, it is reported that ABCA2 downregulation by siRNA decreases Aβ production. 33 The literature findings suggest downregulating ABCA2 to reduce Aβ production as a therapeutic approach to AD therapy. Therefore, it is important to conduct studies testing potential therapeutics in the treatment of AD of downregulating ABCA2.

*ABCA5* : ABCA5 is expressed in many brain regions affected by AD, such as the hippocampus ( Table1 ). In the study conducted by Fu et al (2015), they determined that ABCA5 expression was significantly higher in the hippocampus of individuals with AD compared with the control groups. 34 In the same study, they determined that the decrease in Aβ level in cells transfected with ABCA5 was due to the change in APP processing (not APP expression). 34

*ABCA7* : ABCA7 has mainly expressed in brain microglial cells ( Table 1 ). There is evidence that ABCA7, showing 54% homology with ABCA1, may be involved in AD pathology through Aβ accumulation and lipid metabolism. 35 Studies have reported that *ABCA7* genes are strongly linked to the immune response, cholesterol metabolism, and amyloid hypothesis affected in AD. 36 37 Many investigators suggest that the relationship between ABCA7 and AD pathology is not direct but through lipid metabolism or immune response. There are different opinions regarding the role of ABCA7 in Aβ homeostasis, such as that ABCA7 stimulates the efflux of cholesterol and inhibits Aβ production, and ABCA7 deletion increases the levels of Aβ by changing the clearance of amyloid plaques. ABCA7 has a role in phagocytosis of cytotoxic or antigenic molecules released from cells during apoptosis to maintain tissue homeostasis. 4 Although the underlying mechanisms have not been fully elucidated, it can be predicted that failure to clear phagocytes in apoptotic cells may cause inflammation and thus AD. 38 Studies on the functional role of ABCA7 in AD development show that ABCA7 modulates APP processing. The protective effect of ABCA7 in AD is explained by the fact that ABCA7 expression inhibits Aβ production in cells coexpressing APP, while its suppression causes the opposite effect 39 40 ( Table 1 ). The further cognitive decline that occurs in the cognitive functions of AD patients is explained by some researchers, by the high levels of ABCA7 and by the regulatory function of ABCA7 in phagocyte function. This demonstrates the importance of protein functionality as well as protein aggregation to prevent the progression of AD. 40 41

## ABCB Subfamily

The ABCB subfamily contains both full transporter (four) and semitransporter (seven). ABCB full and half transporters are divided into two groups: ABCB1,4,5,11 and ABCB2,3,6–10, respectively. 10

*ABCB1 (P-glycoprotein, P-gp)* : According to other ABCB family members, ABCB1 is the most studied carrier in CNS. The *ABCB1* gene, which is also expressed in cells in the BBB and identified in neuronal cells, is thought to play a role in the transport of compounds that cannot be transmitted by diffusion to the brain ( Table 1 ). It is thought to serve as the general defense mechanism protecting from the poisoning of potentially harmful lipophilic compounds that could penetrate the carrier BBB. In studies, it is argued that depending on the expression, localization, potency, and multispecificity of this transporter subtype act as an important barrier to the xenobiotics' entrance to the brain. 42 Studies report that ABCB1 is required for normal clearance of Aβ. 43 It is reported that the accumulation of CAA and Aβ increases the risk of developing AD associated with impaired ABCB1 activity. ABCB1, the efflux transporter that prevents various compounds from entering the brain and pumps metabolic waste products from the brain, plays a crucial role in neuroprotection and general CNS homeostasis. 6 Experimental results in Mdrla/b ^−/−^ double knockout mice determined that the brain clearance of Aβ _40_ and Aβ _42_ was significantly lower 30 minutes after intracerebral microinjection of Aβ compared with control animals. Studies report an inverse correlation between Aβ accumulation in the human BBB and vascular P-gp expression in AD. Postmortem study results also report that several cortical brain regions of AD patients have decreased ABCB1 function in the BBB. 44 Consistent with human studies, study findings in mice also show that P-gp expression and function in the BBB is significantly reduced in mice with high Aβ brain levels. In 2005, impaired Aβ clearance was determined in control mice after treatment with ABCB1 inhibitors in ABCB1 knockout mice. Thus, the pathological link between ABCB1 and AD in humans enabled the age-related decline of ABCB1 expression to be identified as ABCB1 as an important Aβ exporter. 7 25 The literature findings highlight the importance of new therapeutic strategies aimed at restoring the BBB ABCB1 expression and function enhancing Aβ clearance and lower Aβ brain levels in AD. 7 45 It is thought that Aβ also plays a role in the intracerebral/intracellular distribution. 46 47 Studies of AD mouse models overexpressing APP lacking ABCB1 show that breast cancer resistance protein (BCRP) function partially compensates for ABCB1-mediated Aβ clearance. It is possible to say that a small decrease in ABCB1 expression and/or activity in the brain plays an important role in causing Aβ deposits due to a decrease in Aβ clearance. Given the correlation between ABCB1 and Aβ, increased clearance of Aβ through ABCB1 upregulation is important for therapeutic approaches developed to slow or stop the progression of AD. In a study of tissue samples taken from the medial temporal lobe, it was reported that ABCB1 expression was inversely proportional to the accumulation of Aβ _40_ and Aβ _42_ . 48 In the study in which wild type and mdr1a ^−^ /b ^−^ knockout mice were injected with ^125^ I-Aβ _40_ and ^125^ I-Aβ _42_ , it was shown that P-gp null animals had significantly higher Aβ _40_ and Aβ _42_ levels in their brains compared with control animals. 44 The study using (R)- ^11^ C verapamil and positron emission tomography in 2012 is the first evidence to demonstrate the direct relationship between AD pathogenesis and decreased ABCB1 function. In the same year, the correlation between decreased ABCB1 due to aging was revealed by this research group. 49 In the study using C57BL/6 mice, it was shown that mice exposed to rifampicin and caffeine increased ABCB1 regulation in the BBB (2.7-fold and 1.5-fold, respectively) compared with the control group and the upregulation was correlated with BBB increasing Aβ _40_ clearance. 15 Another evidence that a deficiency in the ABCB1 transporter can lead to an extra accumulation of Aβ in the brain of mice is provided by the study of Wang et al (2006). 50

Loperamide, an opioid with analgesic effect and not crossing the BBB and an ABCB1 substrate, shows a significant antinociception effect when administered to mice with the ABCB1 inhibitor cyclosporine A. ABCB1 has a fairly broad spectrum of compound substrates, from small molecules such as morphine to peptides such as Aβ. 12 Studies showing the physiological importance of ABCB1 in protecting the brain and the difficulties in delivering therapeutic drugs to the CNS, it was demonstrated that ABCB1 substrates such as ivermectin and drugs that do not normally penetrate the brain cause a 5- to 50-fold increase in the brain to plasma levels of drugs in the mdr1 knockout mice without ABCB1. The study using APP/PS1 mice showed that it was determined that the ABCB1 expression of AD mice was downregulated compared with the control group. In the results of the study using huperzine A, which is extracted from *Huperzia serrata* and which is a powerful acetylcholinesterase inhibitor, it has been reported that ABCB1 is a substrate and exhibits neuroprotective properties when used in the treatment of AD by targeting nicotinic and muscarinic receptors. Huperzine increases the brain-to-plasma concentration ratio in ABCB1-deficient mice and that the increase in this distribution may be mediated by ABCB1. 51

## ABCC Subfamily

This subfamily includes 12 full transporters with export activity and one pseudogene is divided into two groups: ABCA1–6,10–12 and ABCA13, respectively. 10 48

*ABCC1 (multidrug resistance protein 1, MRP1)* : There is some evidence in the literature regarding the protective physiological role of ABCC1 from AD. ABCC1 efflux is important in the removal of cerebral Aβ through the BBB. It has been shown that Abcc1 deficiency elevates Aβ _40_ and Aβ _42_ levels in the brains of APP/PS1 mice. In one study, increase in ABCC1 export activity in BBB was associated with a decrease in Aβ accumulation in AD-induced mice. In another study supporting the role of ABCC1 in Aβ clearance, it was reported that treatment with the thiethylperazine, an ABCC1 agonist, significantly reduced cerebral Aβ accumulation. 52 ABCC1 protein expressed in brain cells ( Table 1 ). 53 54 In the study performed in APP/PS1 × ABCB1 ^−/−^ mice, finding a decrease in Aβ brain levels, it was suggested that Mrp1 activation was the cause of decreased Aβ brain levels. 55 The most effective ABC transporter that affects Aβ brain load is ABCC1. Up to 14-fold increase in Aβ _42_ was detected in ABCC1 knockout mice. There are also studies reporting that ABCC1 can reduce amyloid load in APPPS1 mice by up to 80% through functional activation. 52

*ABCC4* : In the literature, there are no studies reporting ABCC4 transport function in brain capillary endothelium or capillary endothelium cells, but it is reported that ABCC4 mRNA is expressed in the choroid plexus. 55 ABCC4 has been reported to mediate the efflux of organic anions, glutathione-, sulfate-, or glucuronate-conjugated drugs. Although the importance of ABCC4 in drug transport in the BBB is not exactly clear, it is thought to contribute to the BBB function by protecting the brain from xenobiotics. 56

## ABCG Subfamily

The ABCG subfamily consists of six half-carriers with a transmembrane domain. The subfamily consists of five half transporters members (ABCG1,2,4,5,8). 11 22

*ABCG1* : ABCG1 that responsible for regulating cellular lipid homeostasis is expressed in brain cells ( Table 1 ). There are a limited number of studies reporting different views on the role of ABCG1 in AD. Coexpression of ABCG1 and APP has been shown to increase secreted levels of Aβ, indicating that ABCG1-mediated regulation of APP traffic supports the potential development of AD. 57 Other researchers have reported that ABCG1 significantly reduced Aβ production in cells expressing APP, suggesting that ABCG1 inhibits AD pathogenesis.

*ABCG2 (BCRP)* : ABCG2 transporters are mostly expressed in the lumen membrane of BBB cells and pericytes, microglia, astrocytes, and neural progenitor ( Table 1 ). 58 Studies examining the function of ABCG2 in AD and Aβ transport report different results. Samples from AD patients showed no changes in ABCG2 protein expression in the hippocampus and another study reported that ABCG2 was not involved in Aβ _42_ transport of ABCG2 in hAPP mice, 59 in contrast to these studies, the expression of ABCG2 mRNA and protein levels compared with control, it has been reported to increase significantly in AD/CAA patients. 60 In a study designed to determine the action of ABCG2 in AD, greater Aβ accumulation was found of ABCG2 knockout animals injected with Aβ than in control mice. This situation was explained by the researchers as ABCG2 may play a role in obstructing Aβ. Given that ABCB1 expression is downregulated while ABCG2 expression is increased in AD brains, studies investigating both transporters in the same brain regions emphasize that carriers are expressed simultaneously. ABCG2 interacts directly with Aβ and has been found to promote the cellular flow of Aβ _40_ and Aβ _42_ in the BBB. 60 61 Although studies in mouse and human AD brains have reported that upregulation of BCRP is effective in reducing Aβ accumulation, 62 63 there are also studies reporting that there is no difference between BCRP levels in healthy and AD individuals. 59

*ABCG4* : ABCG4 protein is expressed in brain cell such as astrocyte and microglia ( Table 1 ). Studies have reported high expression level of ABCG4 in microglial cells located close to senile plaques in the AD brain. A study was determined that ABCG4 was able to export Aβ in ABCB1/ABCG2-deficient mice and its expression in the brain increased. 63 In another study, it has also been shown that ABCG4 expression in microglial cells is significantly upregulated in AD patients. In the study of AD mouse models, it has been reported that ABCG4 activity to eliminate amyloid deposits contributes to Aβ degradation through microglia-mediated phagocytosis and even completes the proposed role for ABCA7. 63

## Conclusion

Although the interaction of many mechanisms in neurodegenerative diseases creates many risk factors, our knowledge about all of these interaction mechanisms or the results of these interactions is still not sufficient. It is almost impossible to investigate possible combinations of mechanisms in humans by trial and error. It is still unclear whether the change in expression and function of ABC members is a lead or an outcome of AD, but it is clear that they play a significant role in AD physiopathology. There is a need for studies that better describe the changes in expression, function and transport functions and mechanisms of ABC transporters. Results from the literature support the regulation of ABC members as curative to ameliorate or prevent the progression of AD. It has a major pharmacological importance to develop drugs targeting carriers, especially because of the strong evidence that ABCB1 and ABCA1 carriers have in AD pathology.

More studies are needed to elucidate the effect of dysfunction or genetic variation that occurs in ABC carriers that are directly or indirectly in AD pathology, on the production of neurotoxic molecules, especially Aβ. Few studies in the literature have focused on changes in AD and Aβ carrier expression. The elaboration of ABC transporter functions with molecular studies is important in the development of treatment strategies targeting ABC carriers in neurodegenerative diseases such as AD.

## References

[1] Jia Y Wang N Zhang Y Xue D Lou H Liu X Alteration in the function and expression of SLC and ABC transporters in the neurovascular unit in Alzheimer's disease and the clinical significance Aging Dis 2020 11 02 390 404 32257549 10.14336/AD.2019.0519 PMC7069460

[2] Seltzer B Is long-term treatment of Alzheimer's disease with cholinesterase inhibitor therapy justified? Drugs Aging 2007 24 11 881 890 17953456 10.2165/00002512-200724110-00001

[3] Abuznait A H Kaddoumi A Role of ABC transporters in the pathogenesis of Alzheimer's disease ACS Chem Neurosci 2012 3 11 820 831 23181169 10.1021/cn300077c PMC3504479

[4] Kim W S Li H Ruberu K Deletion of Abca7 increases cerebral amyloid-β accumulation in the J20 mouse model of Alzheimer's disease J Neurosci 2013 33 10 4387 4394 23467355 10.1523/JNEUROSCI.4165-12.2013 PMC6704948

[5] Hardy J Selkoe D J The amyloid hypothesis of Alzheimer's disease: progress and problems on the road to therapeutics Science 2002 297(5580):353 356 12130773 10.1126/science.1072994

[6] Wolf A Bauer B Hartz A MS ABC transporter and the Alzheimer's disease enigma Front Psychiatry 2012 3 54 22675311 10.3389/fpsyt.2012.00054 PMC3366330

[7] Pahnke J Langer O Krohn M Alzheimer's and ABC transporters–new opportunities for diagnostics and treatment Neurobiol Dis 2014 72(Pt A):54 60 24746857 10.1016/j.nbd.2014.04.001 PMC4199932

[8] Zlokovic B V Clearing amyloid through the blood-brain barrier J Neurochem 2004 89 04 807 811 15140180 10.1111/j.1471-4159.2004.02385.x

[9] Allikmets R Dean M Cloning of novel ABC transporter genes Methods Enzymol 1998 292 116 130 9711550 10.1016/s0076-6879(98)92011-0

[10] Dean M Rzhetsky A Allikmets R The human ATP-binding cassette (ABC) transporter superfamily Genome Res 2001 11 07 1156 1166 11435397 10.1101/gr.184901

[11] Vasiliou V Vasiliou K Nebert D W Human ATP-binding cassette (ABC) transporter family Hum Genomics 2009 3 03 281 290 19403462 10.1186/1479-7364-3-3-281 PMC2752038

[12] Hartz A M Bauer B ABC transporters in the CNS - an inventory Curr Pharm Biotechnol 2011 12 04 656 673 21118088 10.2174/138920111795164020

[13] Kim W S Weickert C S Garner B Role of ATP-binding cassette transporters in brain lipid transport and neurological disease J Neurochem 2008 104 05 1145 1166 17973979 10.1111/j.1471-4159.2007.05099.x

[14] Gil-Martins E Barbosa D J Silva V Remião F Silva R Dysfunction of ABC transporters at the blood-brain barrier: role in neurological disorders Pharmacol Ther 2020 213 107554 32320731 10.1016/j.pharmthera.2020.107554

[15] Qosa H Miller D S Pasinelli P Trotti D Regulation of ABC efflux transporters at blood-brain barrier in health and neurological disorders Brain Res 2015 1628(Pt B):298 316 26187753 10.1016/j.brainres.2015.07.005 PMC4681613

[16] Saunders N R Habgood M D Møllgaård K Dziegielewska K M The biological significance of brain barrier mechanisms: help or hindrance in drug delivery to the central nervous system?F1000Res 2016;5:F1000 Faculty Rev 313 10.12688/f1000research.7378.1 PMC4786902 26998242

[17] de Boer A G van der Sandt I C Gaillard P J The role of drug transporters at the blood-brain barrier Annu Rev Pharmacol Toxicol 2003 43 629 656 12415123 10.1146/annurev.pharmtox.43.100901.140204

[18] Löscher W Potschka H Blood-brain barrier active efflux transporters: ATP-binding cassette gene family NeuroRx 2005 2 01 86 98 15717060 10.1602/neurorx.2.1.86 PMC539326

[19] Bell R D Zlokovic B V Neurovascular mechanisms and blood-brain barrier disorder in Alzheimer's disease Acta Neuropathol 2009 118 01 103 113 19319544 10.1007/s00401-009-0522-3 PMC2853006

[20] Weiss N Miller F Cazaubon S Couraud P O The blood-brain barrier in brain homeostasis and neurological diseases Biochim Biophys Acta 2009 1788 04 842 857 19061857 10.1016/j.bbamem.2008.10.022

[21] *Dictyostelium* Sequencing Consortium Anjard C Loomis W F Evolutionary analyses of ABC transporters of *Dictyostelium discoideum* Eukaryot Cell 2002 1 04 643 652 12456012 10.1128/EC.1.4.643-652.2002 PMC117992

[22] Dean M Hamon Y Chimini G The human ATP-binding cassette (ABC) transporter superfamily J Lipid Res 2001 42 07 1007 1017 11441126

[23] Mack J T Townsend D M Beljanski V Tew K D The ABCA2 transporter: intracellular roles in trafficking and metabolism of LDL-derived cholesterol and sterol-related compounds Curr Drug Metab 2007 8 01 47 57 17266523 10.2174/138920007779315044 PMC6361144

[24] Gosselet F Candela P Sevin E Berezowski V Cecchelli R Fenart L Transcriptional profiles of receptors and transporters involved in brain cholesterol homeostasis at the blood-brain barrier: use of an in vitro model Brain Res 2009 1249 34 42 18996096 10.1016/j.brainres.2008.10.036

[25] Wahrle S E Jiang H Parsadanian M ABCA1 is required for normal central nervous system ApoE levels and for lipidation of astrocyte-secreted ApoE J Biol Chem 2004 279 39 40987 40993 15269217 10.1074/jbc.M407963200

[26] APOE and Alzheimer Disease Meta Analysis Consortium Farrer L A Cupples L A Haines J L Effects of age, sex, and ethnicity on the association between apolipoprotein E genotype and Alzheimer disease. A meta-analysis JAMA 1997 278 16 1349 1356 9343467

[27] Holtzman D M Fagan A M Mackey B Apolipoprotein E facilitates neuritic and cerebrovascular plaque formation in an Alzheimer's disease model Ann Neurol 2000 47 06 739 747 10852539

[28] Wahrle S E Jiang H Parsadanian M Deletion of Abca1 increases Abeta deposition in the PDAPP transgenic mouse model of Alzheimer disease J Biol Chem 2005 280 52 43236 43242 16207708 10.1074/jbc.M508780200

[29] Broccardo C Nieoullon V Amin R ABCA2 is a marker of neural progenitors and neuronal subsets in the adult rodent brain J Neurochem 2006 97 02 345 355 16539677 10.1111/j.1471-4159.2006.03714.x

[30] Shawahna R Uchida Y Declèves X Transcriptomic and quantitative proteomic analysis of transporters and drug metabolizing enzymes in freshly isolated human brain microvessels Mol Pharm 2011 8 04 1332 1341 21707071 10.1021/mp200129p

[31] Pahnke J Wolkenhauer O Krohn M Walker L C Clinico-pathologic function of cerebral ABC transporters - implications for the pathogenesis of Alzheimer's disease Curr Alzheimer Res 2008 5 04 396 405 18690837 10.2174/156720508785132262 PMC2753594

[32] Chen Z J Vulevic B Ile K E Association of ABCA2 expression with determinants of Alzheimer's disease FASEB J 2004 18 10 1129 1131 15155565 10.1096/fj.03-1490fje

[33] Michaki V Guix F X Vennekens K Down-regulation of the ATP-binding cassette transporter 2 (Abca2) reduces amyloid-β production by altering Nicastrin maturation and intracellular localization J Biol Chem 2012 287 02 1100 1111 22086926 10.1074/jbc.M111.288258 PMC3256850

[34] Fu Y Hsiao J H Paxinos G Halliday G M Kim W S ABCA5 regulates amyloid-β peptide production and is associated with Alzheimer's disease neuropathology J Alzheimers Dis 2015 43 03 857 869 25125465 10.3233/JAD-141320

[35] Quazi F Molday R S Differential phospholipid substrates and directional transport by ATP-binding cassette proteins ABCA1, ABCA7, and ABCA4 and disease-causing mutants J Biol Chem 2013 288 48 34414 34426 24097981 10.1074/jbc.M113.508812 PMC3843056

[36] Iwamoto N Abe-Dohmae S Sato R Yokoyama S ABCA7 expression is regulated by cellular cholesterol through the SREBP2 pathway and associated with phagocytosis J Lipid Res 2006 47 09 1915 1927 16788211 10.1194/jlr.M600127-JLR200

[37] Cascorbi I Flüh C Remmler C Association of ATP-binding cassette transporter variants with the risk of Alzheimer's disease Pharmacogenomics 2013 14 05 485 494 23556446 10.2217/pgs.13.18

[38] Zhao Q F Yu J T Tan M S Tan L ABCA7 in Alzheimer's disease Mol Neurobiol 2015 51 03 1008 1016 24878767 10.1007/s12035-014-8759-9

[39] Chan S L Kim W S Kwok J B ATP-binding cassette transporter A7 regulates processing of amyloid precursor protein in vitro J Neurochem 2008 106 02 793 804 18429932 10.1111/j.1471-4159.2008.05433.x

[40] Bamji-Mirza M Li Y Najem D Genetic variations in ABCA7 can increase secreted levels of amyloid-40 and amyloid-42 peptides and ABCA7 transcription in cell culture models J Alzheimers Dis 2016 53 03 875 892 27314524 10.3233/JAD-150965

[41] Vasquez J B Fardo D W Estus S ABCA7 expression is associated with Alzheimer's disease polymorphism and disease status Neurosci Lett 2013 556 58 62 24141082 10.1016/j.neulet.2013.09.058 PMC3863933

[42] Begley D J ABC transporters and the blood-brain barrier Curr Pharm Des 2004 10 12 1295 1312 15134482 10.2174/1381612043384844

[43] Chai A B Leung G KF Callaghan R Gelissen I C P-glycoprotein: a role in the export of amyloid-β in Alzheimer's disease? FEBS J 2020 287 04 612 625 31750987 10.1111/febs.15148

[44] Cirrito J R Deane R Fagan A M P-glycoprotein deficiency at the blood-brain barrier increases amyloid-beta deposition in an Alzheimer disease mouse model J Clin Invest 2005 115 11 3285 3290 16239972 10.1172/JCI25247 PMC1257538

[45] Hartz A M Miller D S Bauer B Restoring blood-brain barrier P-glycoprotein reduces brain amyloid-beta in a mouse model of Alzheimer's disease Mol Pharmacol 2010 77 05 715 723 20101004 10.1124/mol.109.061754 PMC2872973

[46] Rajagopal A Simon S M Subcellular localization and activity of multidrug resistance proteins Mol Biol Cell 2003 14 08 3389 3399 12925771 10.1091/mbc.E02-11-0704 PMC181575

[47] Donovan M H Yazdani U Norris R D Games D German D C Eisch A J Decreased adult hippocampal neurogenesis in the PDAPP mouse model of Alzheimer's disease J Comp Neurol 2006 495 01 70 83 16432899 10.1002/cne.20840

[48] Vogelgesang S Warzok R W Cascorbi I The role of P-glycoprotein in cerebral amyloid angiopathy; implications for the early pathogenesis of Alzheimer's disease Curr Alzheimer Res 2004 1 02 121 125 15975076 10.2174/1567205043332225 PMC2763541

[49] van Assema D M Lubberink M Boellaard R P-glycoprotein function at the blood-brain barrier: effects of age and gender Mol Imaging Biol 2012 14 06 771 776 22476967 10.1007/s11307-012-0556-0 PMC3492696

[50] Wang Y J Zhou H D Zhou X F Clearance of amyloid-beta in Alzheimer's disease: progress, problems and perspectives Drug Discov Today 2006 11(19-20):931 938 16997144 10.1016/j.drudis.2006.08.004

[51] Damar U Gersner R Johnstone J T Schachter S Rotenberg A Huperzine A as a neuroprotective and antiepileptic drug: a review of preclinical research Expert Rev Neurother 2016 16 06 671 680 27086593 10.1080/14737175.2016.1175303

[52] Krohn M Lange C Hofrichter J Cerebral amyloid-β proteostasis is regulated by the membrane transport protein ABCC1 in mice J Clin Invest 2011 121 10 3924 3931 21881209 10.1172/JCI57867 PMC3195473

[53] Soontornmalai A Vlaming M L Fritschy J M Differential, strain-specific cellular and subcellular distribution of multidrug transporters in murine choroid plexus and blood-brain barrier Neuroscience 2006 138 01 159 169 16361063 10.1016/j.neuroscience.2005.11.011

[54] Roberts L M Black D S Raman C Subcellular localization of transporters along the rat blood-brain barrier and blood-cerebral-spinal fluid barrier by in vivo biotinylation Neuroscience 2008 155 02 423 438 18619525 10.1016/j.neuroscience.2008.06.015

[55] Warren M S Zerangue N Woodford K Comparative gene expression profiles of ABC transporters in brain microvessel endothelial cells and brain in five species including human Pharmacol Res 2009 59 06 404 413 19429473 10.1016/j.phrs.2009.02.007

[56] Sodani K Patel A Kathawala R J Chen Z S Multidrug resistance associated proteins in multidrug resistance Chin J Cancer 2012 31 02 58 72 22098952 10.5732/cjc.011.10329 PMC3777468

[57] Tansley G H Burgess B L Bryan M T The cholesterol transporter ABCG1 modulates the subcellular distribution and proteolytic processing of beta-amyloid precursor protein J Lipid Res 2007 48 05 1022 1034 17293612 10.1194/jlr.M600542-JLR200

[58] Doyle L A Yang W Abruzzo L V A multidrug resistance transporter from human MCF-7 breast cancer cells Proc Natl Acad Sci U S A 1998 95 26 15665 15670 9861027 10.1073/pnas.95.26.15665 PMC28101

[59] Wijesuriya H C Bullock J Y Faull R L Hladky S B Barrand M A ABC efflux transporters in brain vasculature of Alzheimer's subjects Brain Res 2010 1358 228 238 20727860 10.1016/j.brainres.2010.08.034

[60] Xiong H Callaghan D Jones A ABCG2 is upregulated in Alzheimer's brain with cerebral amyloid angiopathy and may act as a gatekeeper at the blood-brain barrier for Abeta(1-40) peptides J Neurosci 2009 29 17 5463 5475 19403814 10.1523/JNEUROSCI.5103-08.2009 PMC2745912

[61] Zeng Y Callaghan D Xiong H Yang Z Huang P Zhang W Abcg2 deficiency augments oxidative stress and cognitive deficits in Tg-SwDI transgenic mice J Neurochem 2012 122 02 456 469 22578166 10.1111/j.1471-4159.2012.07783.x

[62] Shen S Callaghan D Juzwik C Xiong H Huang P Zhang W ABCG2 reduces ROS-mediated toxicity and inflammation: a potential role in Alzheimer's disease J Neurochem 2010 114 06 1590 1604 20626554 10.1111/j.1471-4159.2010.06887.x

[63] Do T M Noel-Hudson M S Ribes S ABCG2- and ABCG4-mediated efflux of amyloid-β peptide 1-40 at the mouse blood-brain barrier J Alzheimers Dis 2012 30 01 155 166 22391220 10.3233/JAD-2012-112189

